# Rapidly destructive osteoarthritis of the hip joint: a case series

**DOI:** 10.1186/1749-799X-3-3

**Published:** 2008-01-11

**Authors:** Sameer Batra, Meenakshi Batra, A McMurtrie, AK Sinha

**Affiliations:** 1 Division of spine surgery, Department Of Orthopaedics, Llandough Hospital, Penarth, UK; 2 Department of pathology, Wrexham Maelor hospital, Wrexham, UK; 3 Department Of Orthopaedics, Gwynedd Hospital, North West Wales NHS Trust, Bangor, Gwynedd, UK

## Abstract

**Background:**

Rapidly destructive arthrosis of the hip is a rare and incompletely understood disorder with scarce literature about variations in natural history within a population.

**Methods:**

A series of cases from North Wales with rapid progressive joint destruction and extensive subchondral bone loss in the femoral head and acetabulum are presented. Radiographic findings mimicked those of other disorders such as septic arthritis, rheumatoid and seronegative arthritis, primary osteonecrosis with secondary osteoarthritis, or neuropathic osteoarthropathy, but none of the patients had clinical, pathologic, or laboratory evidence of these entities.

**Results:**

Rapid progression of hip pain and disability was a consistent clinical feature. The average duration of symptoms was 1.4 years. Radiographs obtained at various intervals before surgery (average 14 months) in 18 patients documented rapid hip destruction, involvement being unilateral in 13 cases. All patients underwent total hip arthroplasty, and osteoarthritis was confirmed at pathologic examination.

**Conclusion:**

The authors postulate that these cases represent an uncommon subset of osteoarthritis and regular review, both clinically and radiologically, are required to assess speed of progression and prevent rapid loss of bone stock without the surgeon being aware. These cases are unsuitable for being placed on long waiting list due to technical difficulties in delayed surgery and compromised outcome following surgery.

## Background

Rapidly progressive hip disease (RDHD) is a rare syndrome of unknown etiology and distinct from ischaemic necrosis of the femoral head, resulting in rapid deterioration of both the femoral and acetabular aspects of the hip joint with disappearance of the femoral head as first reported by Forestier [1]. The natural history of hip osteoarthrosis has been extensively documented [1-3] with well known inter-racial variations [4]. Little, however, has been written about variation in natural history within a population and only scant mention of discrete subgroup of this type appears in the English language journals, with studies originating mainly in continental Europe. Attempts to characterise this disorder are limited to case reports where inconsistent associations have been made.

The senior author (AKS) came across a series of cases from North Wales with this rapidly progressive presentation of osteoarthritis and became concerned that these patients may be losing up to 15 mm of bone stock annually while on the waiting list. Failure to act on rapidly worsening hip arthrosis is well recognised to compromise the success of surgery and is clearly undesirable, hence making these unsuitable for being placed on long waiting lists. Moreover, if such cases can be identified, it should be made possible to offer them a priority for treatment.

We hereby present this series of cases from a population group from North Wales that we believe represent a subset of osteoarthritis that has been recognized in the literature as rapid destructive osteoarthritis (RDO) [1-5].

We highlight the importance of repeat radiographs for patients with continued severe hip pain without an apparent cause. The purpose of our report is to make clinicians faced with a diagnostic dilemma in general practice, rheumatology or orthopaedic clinics, aware of the condition and present the clinical, radiographic and pathologic features of this unique hip disorder that can be confused radiographically with other more well recognised destructive hip arthropathies.

## Methods

A retrospective review of patients with a clinical profile and serial radiographs suggestive of a rapidly progressive hip disease was undertaken. The search revealed 145 patients who received I56 primary implants. Hospital notes from all cases were studied and patients with inflammatory arthritis, primary osteonecrosis, renal osteodystrophy and trauma were identified and excluded, leaving 115 patients with primary osteoarthritis including this subset. This revealed 18 patients (incidence of RDHD is about 15.7%) who met our criteria for RDHD in the period between May 2004 and May 2006 carefully excluding inflammatory arthritis, primary osteonecrosis, renal osteodystrophy and trauma. A retrospective analysis of clinical and radiographic records was performed. When available, serial radiographs of the hips were similarly evaluated, and the time between the last normal radiograph and that showing most severe destruction was recorded. The criteria for RDHD were a clinical history of hip pain of 1–6 months duration, a radiologic appearance of rapidly progressive atrophic bone destruction involving the femoral head and the acetabulum, the absence of clinical or laboratory evidence of infectious, neurologic, metabolic, endocrinologic, or inflammatory disorders. This excluded patients who were found to have rheumatoid arthritis, primary avascular necrosis, renal osteodystrophy, abnormalities of calcium metabolism or where trauma was suspected of having played a part in the hip disease. Septic arthritis was excluded on the basis of results of joint aspiration and/or clinical and laboratory examinations. Neurologic disease as well as a history of steroid medication was assessed. Ischemic necrosis was excluded on the basis of radiographic appearance of atrophic destruction and failure to identify the usual stages of osteonecrosis on serial radiographs.

Finally, the surgical findings at joint replacement or biopsy were recorded in cases in which histologic examination had been performed. These data were reviewed.

## Results

**Figure 1:**
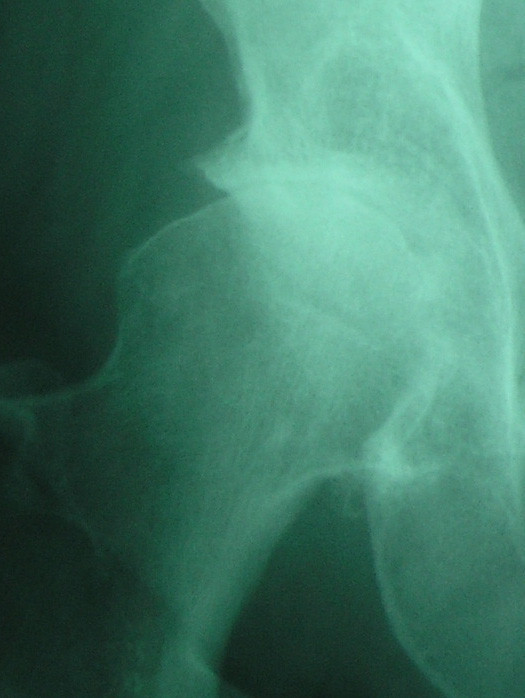
Anteroposterior radiographs obtained in a 67-year-old woman with painful right hip. View obtained 3 months after pain began reveals typical osteoarthritis

**Figure 2:**
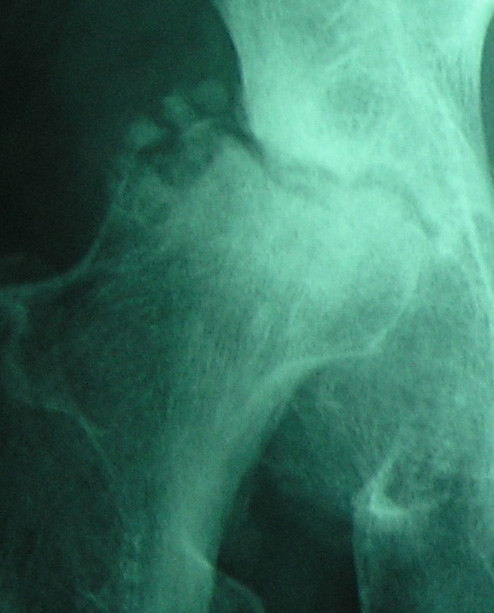
6 months later, severe flattening of the femoral head with an eccentric depression of the lateral articular surface associated with superolateral subluxation, sclerosis, and subchondral defects. Notice the hatchet-like appearance of the femoral head.

**Figure 3:**
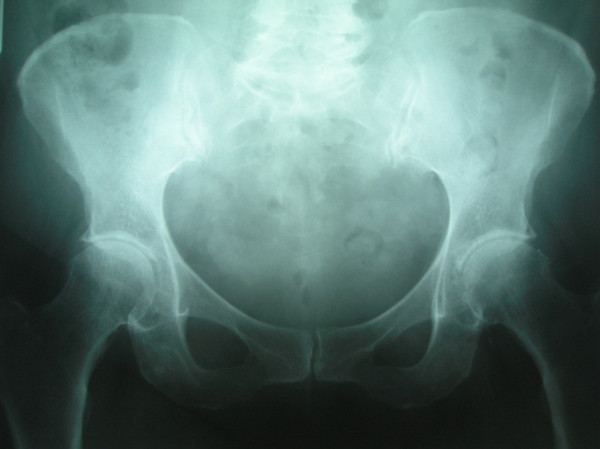
Anteroposterior radiographs obtained in a 70-year-old woman with painful bilateral hips of 4 months duration.

**Figure 4:**
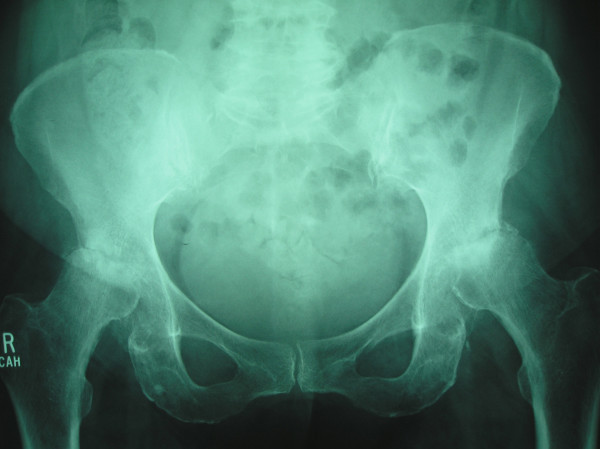
3 months later reveals further worsening with prominent femoral head flattening and marked sclerosis with absence of osteophytes.

**Figure 5:**
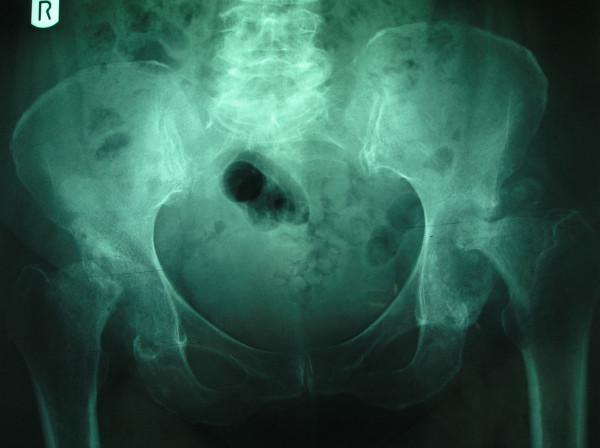
4 months later, severe destruction of femoral heads on both sides.

The study group contained 16 women (89%) and 2 males (11%), average age 68.8 years (range, 47–81 years). The duration of symptoms ranged from 6 months to 3 years (mean, 1.4 years). All patients presented with hip pain. 14 patients (78%) suffered pain for two years or less. None of these18 cases had evidence of rheumatoid arthritis, primary avascular necrosis, renal osteodystrophy, abnormalities of calcium metabolism vascular history, alcoholism, and there was no relevant occupational or pharmacological history. None of the patients underwent systemic steroid administration or injection of steroids to the affected joint, and none had a history of alcohol abuse. The mean time for the radiographic appearance of joint destruction after a negative radiograph was 14 months (range, 4 months to 2 years). There were at least four hips with dramatic destruction over a period of 6 months. (Two examples illustrated in Figures 1, 2, 3, 4, 5)

The femoral heads were small, and in most cases, the weight-bearing surface was flat. Resected femoral heads showed disappearance of articular cartilage in the weight-bearing area and a large part of the subchondral bone was destroyed. The articular surface was eroded and flattened with some ebumation and the presence of fibrous tissues. In cases with severe destruction, the articular surface was totally covered by fibrous tissues.

Histology was available in 11 cases. Histologic examination failed to demonstrate evidence of acute or chronic inflammation or any pathologic abnormality other than severe degenerative changes. Areas of segmental osteonecrosis were present at the subarticular region in 4 cases. Necrotic bone and bone marrow were evident. Histology of femoral heads failed to show the findings typical of primary osteonecrosis, in which trabecular and marrow necrosis (necrotic zone) is observed adjacent to viable bone marrow (viable zone) with an intervening zone of repair (reparative zone).

There was no evidence of sepsis in any of the specimens analysed, and over the course of this study none of the study group presented with evidence of deep sepsis associated with prosthesis.

## Discussion

Rapidly destructive arthrosis of the hip an uncommon subset of osteoarthritis and was first described by Forestier in 1957 [1] and subsequently labelled atrophic osteoarthritis, rapidly progressive osteoarthritis, destructive osteoarthritis, and Postel's osteoarthritis [2]. Lequesne defined it as a greater than 2 mm/year rate of joint space narrowing, i.e. loss of more than 50% of the joint space within 1 year [5].

Over the next three decades, subsequent series and reports emphasized the clinical, radiographic and pathologic findings of this unusual condition. The series of cases in our region with rapid severe progressive hip joint destruction within few months drew our attention, which we think need to be prioritised with regard to surgical intervention and are unsuitable for un-reviewed placement on long waiting lists. This is mainly due to the intraoperative technical difficulty due to significant acetabular bone loss; increased blood loss during surgery coupled with increased operating times and need for special implants all making joint reconstruction challenging in these patients. This is associated with compromised outcome following surgery and researchers have indicated that overall results and survivorship are adversely affected by any acetabular defect, and this is certainly the main technical problem with rapidly progressive cases [6]. There is therefore clearly a need for early diagnosis of this rapidly destructive hip OA.

Initially, radiographs show either normal anatomy or mild osteoarthritic changes. Follow-up radiographs in a few months of the onset of symptoms, demonstrate destruction of the femoral head and acetabulum with sclerosis, subchondral cysts, and minimal or no osteophytes. RDHD may radiographically be confused with other disease entities such as primary osteonecrosis with secondary arthritis, rheumatoid arthritis, neuropathic arthropathy or septic arthritis. In most cases, however, clinical history and radiographic findings are sufficient for excluding the latter entities. However, the rapid progression of this disease makes it difficult to obtain sequential radiographs in its early stages [4].

The average age at onset is greater than that of patients with ordinary coxarthrosis with primarily unilateral involvement, and severe pain but relatively preserved range of motion. One characteristic of rapidly progressive cases, however, is that they appear radiologically to be atrophic rather than hypertrophic with an almost complete absence of osteophytes [7]. They are also characterized by the presence of lateral disease [8,9]. Overweight, elderly women with lateral disease and minimal osteophyte formation appear to represent the group most "at risk" [9].

The pathologic findings, however, are consistent with osteoarthritis. Our study supports the features of RDHD described previously in literature. The short duration of symptoms as well as the retrospective nature of study may explain the absence of sequential radiographs and MR imaging in our patients. The referring clinician in primary care may not be aware of this entity and as the patient is already on waiting list for hip replacement, repeat radiographs may not be deemed necessary. But it has to be emphasized that patients suffering rapid deterioration cannot reliably be picked up by questionnaire and do not always alert the clinicians to the worsening symptoms [10]. In addition, x-ray and pathologic progression may approach [11], but not exactly match pain and disability [10]. If the clinician is made aware of RDHD, the need for extensive investigation to exclude sepsis may be obviated. Furthermore, placement of a total joint prosthesis in a patient with neuropathy can often lead to failure [2].

The precise pathogenesis of rapidly destructive hip osteoarthritis remains unclear. Direct drug toxicity and nonsteroidal anti-inflammatory drugs were first incriminated but their contributory effect was subsequently challenged [2,12]. Subchondral bone ischemia and cell necrosis recently have been emphasized as major factors in the development of RDHD by Mitrovic and Riera [13]. Pathologically, many investigators have reported that osteoclast count is significantly greater in active areas of the hip in RDHD than in osteoarthritic patients along with vascular-rich granulation, suggest ing that vascularity is an important factor in the osteolysis and destruction of the bone [14,15]. Others have demonstrated elevated levels of interleukin-6 (IL-6), IL-1β in the joint fluid of affected patients as well as increased secretion of matrix metalloproteinases(MMP) by fibroblasts from the affected synovium and subchondral cysts [16,17]. Although implicated as a causative factor, no genetic analysis such as that using human leukocyte antigen for rapidly destructive arthropathy has been reported.

Histologically, partial necrosis of the subchondral bone has been recognized, but it is unknown whether osteonecrosis is the primary cause of RDHD or secondary to degenerative changes because it is difficult to determine the pathogenesis from late stage specimens obtained at total hip arthroplasty. Histologically, RDHD has elements characteristic of ordinary coxarthrosis or idiopathic avascular necrosis (AVN) of the femoral head (or both): Destruction of the femoral head and the acetabulum is severe and widespread, articular cartilage disappears completely, and the synovial membrane is hypervasculated and slightly inflamed. However, no new bone formation has been observed and no osteophytes have been found, which are typical of ordinary coxarthrosis. Findings such as more rapid and more pervasive invasion, lack of a line of demarcation between necrotic and healthy tissue, and lack of a recurrent necrosis differentiate the disease from idiopathic AVN of the femoral head. Immunohistochemical analysis of articular cartilage and the synovium also demonstrates a different pattern from that of idiopathic AVN and coxarthrosis. However, the pattern is similar to that seen in rheumatoid arthritis. This immunologic response of the articular cartilage explains the chondrolysis associated with the disease, which is usually identified roentogenographically in its early stage [18].

The histologic features observed in our series – nonspecific severe degenerative changes with few marginal osteophytes and no evidence of primary osteonecrosis, pannus formation, and crystal deposition are similar to those observed by previous authors [13,19,20]. The eburnated areas showed partial osteonecrosis, which was consistent with secondary osteonecrosis in osteoarthritis [19]. Furthermore, none of our patients exhibited chondrocalcinosis (pelvis, knees) radiographically or histopathologically. Moreover, pathologic confirmation of osteoarthritis was available in 9 cases.

MR imaging can be valuable in the evaluation of such disorders. Therefore, radiologists should be aware of MR imaging findings in patients with rapidly destructive hip osteoarthritis that can overlap other diagnostic entities. The key MR imaging features include an extensive bone marrow edema like pattern in the femoral head and neck, femoral head flattening, and cyst like subchondral defects.

Our study has a major limitation for being a retrospective study with an inherent selection bias and the study group is already selected by virtue of having come to the need for a hip replacement. However, distinction of RDO from an infectious process or neuropathic osteoarthropathy is of paramount importance and familiarity with this entity may obviate unnecessary diagnostic tests and lengthy delay in treatment with total hip replacement.

## Conclusion

In conclusion, this form has been considered by different authors as a variant of OA, a subset of OA, a severe form of OA, an inflammatory phase of OA and an entity distinct from OA. This study confirmed the radiological definition and the clinical features of this condition and demonstrated the reliability of the pathology examination of the femoral head and the articular capsule. We should aim therefore, to identify such cases early and give them priority. A rational approach would be to ensure that orthopaedic surgeons, trainees and primary care clinicians are aware of this entity and to emphasize on the importance of repeat radiographs for patients with continued severe hip pain without an apparent cause. The decisions about the need for surgery and the selection of cases should be made purely on clinical grounds and not on their rank in the waiting lists.

## Competing interests

'The authors declare that they have no competing interests'. We have not have you received reimbursements, fees, funding, or salary from an organization that may in any way gain or lose financially from the publication of this manuscript, either now or in the future ;in the past five years.

## Authors' contributions

SB conceived the study, carried out the data collection, drafted the manuscript. AM carried out the data collection and helped in preparation of manuscript; M participated in the manuscript preparation and reviewing the literature. AKS participated in the design and coordination and helped to draft the manuscript and revising it. All authors read and approved the final manuscript.
